# Device-assessed sleep health among older patients with heart failure: a cross-sectional study using actigraphy

**DOI:** 10.1136/bmjopen-2025-111622

**Published:** 2026-02-02

**Authors:** Sunanthiny Krishnan, Shelley Taylor, Charlotte L Edwardson, Alex V Rowlands, Iain B Squire, Shirley Sze

**Affiliations:** 1 NIHR Leicester Biomedical Research Centre, Leicester, England, UK; 2 Department of Cardiovascular Sciences, University of Leicester, Leicester, England, UK; 3 Diabetes Research Centre, University of Leicester, Leicester, England, UK; 4 Alliance for Research in Exercise, Nutrition and Activity (ARENA), UniSA Allied Health and Human Performance, University of South Australia, Adelaide, South Australia, Australia

**Keywords:** Heart failure, SLEEP MEDICINE, Aged, Frail Elderly, Wearable Devices

## Abstract

**Abstract:**

**Objective:**

Poor sleep is common among patients with heart failure (HF) and is associated with adverse cardiovascular outcomes. The utility of actigraphy in sleep assessment, especially among older adults, remains underexplored. This study aimed to assess sleep health among older adults with HF using actigraphy and explore associations between sleep parameters and cardiac biomarkers, functional performance and quality of life (QoL).

**Design:**

A cross-sectional study.

**Setting:**

The study was conducted at an outpatient HF clinic within a tertiary cardiology service in a National Health Service hospital in the UK between March and October 2023.

**Participants:**

A total of 150 older adults aged ≥65 years with a diagnosis of HF were enrolled.

**Methods:**

Participants were given a wrist-accelerometer to wear for 7 days. On Day 0, patients completed a 4-metre walk test (4MWT), handgrip strength test (HGST), Timed Up and Go test (TUGT), Barthel Index (BI), Kansas City Cardiomyopathy Questionnaire (KCCQ-12) and frailty assessment (Clinical Frailty Scale, CFS). Subsequently, they were fitted with an accelerometer, with the device configured to start recording the following day (Day 1). Sleep outcomes were calculated after a 7-day wear period and averaged across valid nights (minimum 3 nights of recording, noon-to-noon with ≥16 hours wear-time). Sleep parameters studied include average sleep efficiency, sleep period time window, sleep duration, sleep onset and wake up time, wake after sleep onset (WASO), sleep interruptions and Sleep Regularity Index (SRI). Inefficient sleep was defined as sleep efficiency <80%. Regression analysis was used to examine associations between sleep parameters and the previously stated tests and assessments, adjusting for age, gender and comorbidities.

**Outcome measures:**

The primary outcome measure was sleep efficiency; all other sleep parameters were classified as secondary or exploratory outcomes.

**Results:**

Accelerometry data from 145 participants were analysed; 42% had inefficient sleep based on average sleep efficiency across valid nights. These patients had significantly higher plasma N-terminal pro-B-type natriuretic peptide (NT-proBNP) levels (p=0.044). No statistically significant difference was noted in 4MWT, HGST, TUGT, BI, KCCQ-12 and CFS between patients with sleep efficiency <80% and those with sleep efficiency ≥80%. Lower sleep efficiency was associated with lower BI scores (adjusted β=0.271, p=0.016) and worse frailty (adjusted β=−0.017, p=0.014). Lower SRI was associated with worse New York Heart Association class (adjusted β=−0.009, p=0.007), BI scores (adjusted β=0.310, p<0.001), frailty (adjusted β=−0.017, p<0.001) and QoL (adjusted β=0.344, p=0.001); longer WASO was associated with slower gait speed (adjusted β=−0.039, p=0.040).

**Conclusions:**

Older adults with HF who had inefficient sleep had significantly higher NT-proBNP levels. Lower sleep efficiency was associated with higher functional dependence and frailty. Sleep irregularity was linked to HF symptom load, frailty, functional performance and QoL, while sleep fragmentation was associated with impaired gait speed.

**STRENGTHS AND LIMITATIONS OF THIS STUDY**

- In this study, accelerometry was used for objective assessment of sleep to overcome the risk of recall bias associated with retrospective self-reported instruments.
- A wide range of sleep outcomes was explored, facilitating nuanced understanding of sleep patterns and their effect on older people with heart failure.
- The cross-sectional design of the study precludes inference on causality.
- Sample size is limited and based on a single population; therefore, the observations may not be generalisable.

## Introduction

 Sleep health refers to a multidimensional pattern of sleep–wakefulness that promotes physical and mental well-being within the context of individual, social and environmental demands.^1^ Commonly anchored on constructs such as duration, quality, regularity and efficiency, sleep health is a key determinant of cardiovascular health.^2^ Extensive evidence indicates an association between poor sleep health and cardiovascular disease (CVD).^3 4^ Sleep duration, both short and long, is linked to increased risk of cardiovascular outcomes and all-cause mortality.^5^,^7^ A meta-analysis of 17 cohort studies with over 311 000 participants reports that insomnia, defined as the subjective feeling of non-restorative sleep including difficulty initiating or maintaining sleep, is associated with 33% higher risk of CVD mortality.^8^ Among patients living with heart failure (HF), poor sleep quality is high with a reported prevalence between 50% and 80%.^9^,^12^ Restless sleep, difficulty initiating and maintaining sleep, poor sleep continuity and early morning awakening are commonly reported sleep disturbances in this clinical cohort.^9 13 14^

The wearing effect of sleep deprivation on quality of life (QoL) of patients with HF is well documented. Decline in sleep quality has also been shown to impair functional performance, including activities of daily living (ADL), social independence and cognitive functioning.^14 15^ The aetiology of sleep disruption in patients with HF is rarely singular but often a composite of multiple factors. Symptoms of congestion such as paroxysmal nocturnal dyspnoea and orthopnoea are among the most frequently cited causes, alongside nocturia.^16^ Sleep disordered breathing, specifically obstructive sleep apnoea, and central sleep apnoea with Cheyne-Stokes breathing, are also implicated in HF populations irrespective of phenotype, with prevalence up to 60%.^17^ Interestingly, daytime somnolence, typically a result of poor night-time sleep, is independently associated with increased risk of cardiovascular mortality.^18 19^

In recent years, there has been a blooming interest in the assessment of sleep health among patients with HF. However, few studies have adopted device-based assessment of sleep with even fewer specifically focussing on older people with HF. In most cases, these studies rely on retrospective, self-reported sleep using questionnaires such as the Pittsburgh Sleep Quality Index (PSQI),^20^ Athens Insomnia Scale^21^ and Insomnia Severity Index (ISI).^22^ These subjective measures require a long recall period, ranging from 2 to 4 weeks, which arguably challenges the reliability of the results,^23^ particularly in older people.

While polysomnography (PSG) remains the gold standard for assessment of sleep, the procedure is costly and intrusive to patients’ normal sleep routine.^24^ Actigraphy-based sleep assessment is a feasible, objective and non-invasive alternative allowing monitoring of patients’ sleep pattern in their natural environment. Additionally, actigraphy allows collection of sleep data over an extended period of time (days or weeks) which can provide more reliable estimates of sleep parameters compared with PSG, which is typically performed for only one or two nights.^25^ Importantly, the validity of actigraphy-based sleep evaluation has been established against PSG, with an estimated agreement ranging from 91% to 93%.^25 26^

In this study, we sought to summarise sleep health among older patients with HF using actigraphy and explore associations between sleep parameters and cardiac biomarkers, functional performance and health-related QoL (HRQoL).

## Methods

### Study population

In this cross-sectional study, we recruited 150 participants aged ≥65 years with a diagnosis of HF for at least 1 year. Diagnosis of HF was established based on the presence of HF symptoms (eg, breathlessness, pedal oedema and reduced effort tolerance), echocardiographic evidence of cardiac dysfunction and/or raised natriuretic peptide levels.^27^ Patients with documented cognitive impairment, history of hospitalisation within the past 2 weeks and recent wrist injury of the non-dominant arm (where the accelerometer is worn) were excluded. Recruitment took place at an outpatient HF clinic within a tertiary cardiology service in a National Health Service hospital in the UK between March and October 2023. Convenience sampling was used to recruit participants during the fixed study period. A formal sample size calculation was not undertaken due to the exploratory nature of the study.

### Data collection

#### Overview

All baseline assessments were conducted on Day 0. Participants were fitted with the accelerometer after completing the baseline assessments, with the device configured to start recording the following day, Day 1. Participants were instructed to wear the device for 7 consecutive days, 24 hours/day. Sleep data were downloaded from the devices after the 7-day wear period for analysis.

### Baseline assessments

All patients were reviewed by a cardiologist on the day of enrolment. HF symptom burden was ascertained using the New York Heart Association (NYHA) Functional Classification. All patients had an echocardiogram to assess left ventricular ejection fraction (LVEF) and blood tests including N-terminal pro-B-type natriuretic peptide (NT-proBNP, ng/L) levels, full blood count and renal function. Patients with LVEF <40% were categorised as having HF with reduced ejection fraction (HFrEF), 41–49% as HF with mildly reduced ejection fraction (HFmrEF) and 50% or greater as HF with preserved ejection fraction (HFpEF).^27^

Patients underwent several additional assessments, including evaluation of functional performance (ie, usual gait speed, handgrip strength, dynamic balance, ADL), frailty status and HRQoL.

#### Usual gait speed

Participants’ gait speed was assessed using the standardised 4-metre walk test.^28^ Patients were instructed to walk from a standing start at their usual pace on a flat 4 m course and timed using a stopwatch. Participants were allowed to use their usual assistive devices during the test and the average reading of two attempts was used for analysis. Gait speed (m/sec) was computed as distance (4 m) divided by the average time taken to complete the course (seconds). Higher speed corresponds to better functional mobility.

#### Handgrip strength

Handgrip strength, a surrogate for muscle strength,^29^ was assessed using an analogue Smedley spring dynamometer. Participants’ maximum-effort isometric contraction was measured three times using their dominant hand, with a brief period to rest between each trial. The maximum reading of the three trials was taken for analysis. Grip strength is expressed in kilograms; higher value indicates greater muscle strength.

#### Dynamic balance

The Timed Up and Go Test is a validated instrument that assesses mobility and balance in older adults.^30^ Participants were instructed to stand up from a chair of a standard height, walk a distance of 3 m at their usual pace, turn around, return to the chair and sit down. They were allowed to use their usual walking aids but no other physical assistance was given during the test. Participants began the task seated on a chair with their backs and arms resting. Timing commenced when participants attempted to stand up from the chair and stopped when they returned to the seated position. A stopwatch was used to measure the time. The test is scored in terms of time (seconds) taken to complete the task; shorter completion time correlates with better physical performance.

#### Activities of daily living

Functional capacity in performing ADL was measured using the Barthel Index (BI),^31^ which comprises 10 domains of ADL, each scored on an ordinal scale based on the patient’s physical independence. Patients and/or their accompanying caregivers were interviewed on the former’s ability to perform each of the ADL and scored accordingly. With a total score ranging from 0 to 100, higher scores reflect better ability to perform ADLs independently.

#### Frailty

Baseline frailty status was determined using the Clinical Frailty Scale (CFS),^32^ which categorises degree of frailty on a nine-point scale based on patient’s global function and cognition, with 1 being very fit and 9, terminally ill. Patients with a CFS score ≥5 were deemed frail.

#### Health-related quality of life

Kansas City Cardiomyopathy Questionnaire (KCCQ-12), a HF-specific HRQoL measure, was used to assess the impact of HF on patients’ health status.^33^ A shorter version of the original 23-item questionnaire, KCCQ-12 retains the psychometric properties of the full instrument and measures health on four domains: physical limitation (3 items), symptom frequency (4 items), QoL (2 items) and social limitations (3 items). An overall summary score was also calculated (average of physical limitation, symptom frequency, QoL and social limitation). The scores range from 0 to 100, with higher scores corresponding to better health status.

### Actigraphy

#### Accelerometer

Patients were fitted with a GENEActiv triaxial accelerometer (ActivInsights, Cambridgeshire, UK), on their non-dominant wrist for 7 days continuously (24 hours/day). Each device was initialised to record at a frequency of 25 Hz. During the device wear period, patients were instructed to complete a sleep diary, recording their light-off time at night and morning wake time. The sleep diary was used to guide the accelerometry algorithm to identify the main sleep period.^34^

#### Accelerometry data processing

Data from the accelerometers were downloaded as ‘bin’ files using the GENEActiv PC software (V.3.2) and analysed using the R-package GGIR V.3.0–9.^35 36^ Signal processing in GGIR includes autocalibration using local gravity as a reference,^37^ detection of sustained abnormally high values, detection of non-wear and calculation of the average magnitude of dynamic acceleration, corrected for gravity averaged over 5 s epochs and expressed in milligravitational units (m*g*). Participants were excluded from analysis if their accelerometer files showed post-calibration error greater than 0.01 g (10 m*g*) and/or they did not have three valid nights of recordings.^38^ One valid night is defined as noon-to-noon with >16 hours wear-time within 24 hours.^34^

In GGIR, sleep is detected based on a period of sustained inactivity bout (SIB), defined as the absence of wrist rotation (z-axis) by more than 5 degrees for longer than 5 min.^36^ As SIB is non-specific and encompasses all episodes of rests during a 24-hour period including daytime sleep and sedentary behaviour, the sleep diary was used to differentiate the main nightly sleep period time (SPT) from other SIBs. Only SIB that occurred during the SPT was classed as sleep. Patients’ self-reported lights-off and wake times were entered manually into the system to guide the algorithm to accurately detect the SPT-window.

Relative to PSG, the accuracy and C-statistic for SPT-window classification by GENEActiv accelerometer has been reported to be 87% and 0.86, respectively. The average sensitivity to detect sleep as part of the SPT-window is above 91%.^34^

#### Sleep parameters

Sleep measures studied include sleep efficiency, SPT-window, sleep duration, sleep onset time, wake up time, duration of wake after sleep onset (WASO), number of sleep interruptions and Sleep Regularity Index (SRI). Sleep outcomes were averaged across all valid nights within the 7-day study period. For SRI, the SRI1 outcome in GGIR was used, with calculations based on rolling 30 s epochs. This is modified from the SRI proposed by Phillips *et al*^39^ and Windred *et al*^40^ in that it is calculated per day-pair rather than all valid days. Further details on SRI processing are described elsewhere.^41^

**Figure 1:**
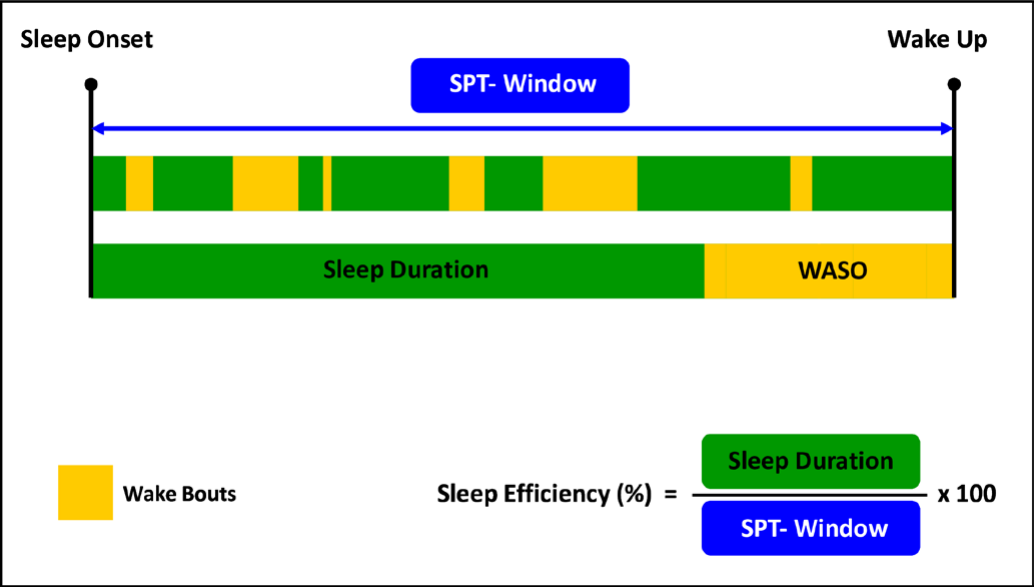
A schematic representation of sleep parameters derived from accelerometry data. SPT-Window, sleep period time window; WASO, wake after sleep onset.

Definitions of each sleep parameter are provided in table 1. Figure 1 illustrates these metrics in the context of an overnight sleep.

**Table 1:** Definition of sleep parameters derived from accelerometry data

Sleep parameters	Definition
SPT-Window	Duration between sleep onset and waking up time (hours)
Sleep onset time	Start of SPT-Window (HH:MM)
Wake up time	End of SPT-Window (HH:MM)
Sleep duration	Duration of actual sleep within SPT-Window discounting any wake time (hours)
Wake after sleep onset	Total duration of wake bouts within the SPT-Window (hours)
Number of sleep interruptions	Number of wake bouts during SPT-Window
Sleep efficiency	(Sleep duration/SPT-Window)× 100 (%)
Sleep Regularity Index^39^	Percentage probability of an individual being in the same state (asleep vs awake) at any two points 24 hours apart (%)

SPT-Window, sleep period time window.

We selected sleep efficiency as the primary outcome as actigraphy-derived sleep efficiency has been shown to positively mirror self-perceived quality of sleep.^42^ Furthermore, sleep efficiency is strongly associated with risk of mortality in older people, with the risk increasing substantially when sleep efficiency is below 80%.^43^ Therefore, in this study, we defined inefficient sleep as sleep efficiency <80%. All other sleep parameters were classified as secondary or exploratory outcomes.

### Statistical analysis

Shapiro-Wilk test indicated that the distribution of sleep data departed from normality. Therefore, non-parametric statistics were used in all analyses. The study population was stratified based on their sleep efficiency, that is, <80% (inefficient sleep) versus ≥80% (efficient sleep). Sleep efficiency was dichotomised to facilitate comparison of clinical characteristics. Comparison of continuous variables between the two categories was analysed using the Mann-Whitney U Test and the χ² test was employed for comparison between categorical variables.

Linear regression models were used to assess the effect of sleep parameters on markers of HF severity, functional status and HRQoL. Sleep efficiency, SPT-Window, WASO, sleep onset time, wake up time, SRI, sleep duration and number of sleep interruptions were selected as independent variables, while markers of HF severity (NT-proBNP, LVEF, NYHA Class), functional status (usual gait speed, handgrip strength, dynamic balance, ADL, frailty) and HRQoL measures (KCCQ-Overall Summary Score, KCCQ-Physical Limitation Score, KCCQ-Symptom Frequency Score, KCCQ-QoL Score and KCCQ-Social Limitation Score) were designated as dependent variables. For all association analyses, sleep efficiency was considered as a continuous variable. Both unadjusted and adjusted regression models were performed, with adjusted models controlling for potential confounders, that is, age, gender and number of comorbidities.

All analyses were performed using SPSS V.28 (SPSS, Chicago, Illinois). A two-tailed p value of <0.05 was considered statistically significant. P values are interpreted descriptively without multiplicity adjustment. A supplementary analysis to account for false discovery rate (FDR) was performed applying the Benjamini-Hochberg procedure,^44^ without further interpretation. The FDR threshold was set at 0.05.

### Patient and public involvement

Patients or the public were not involved in the design, or conduct, or reporting, or dissemination plans of this research.

## Results

### Study participants

**Figure 2:**
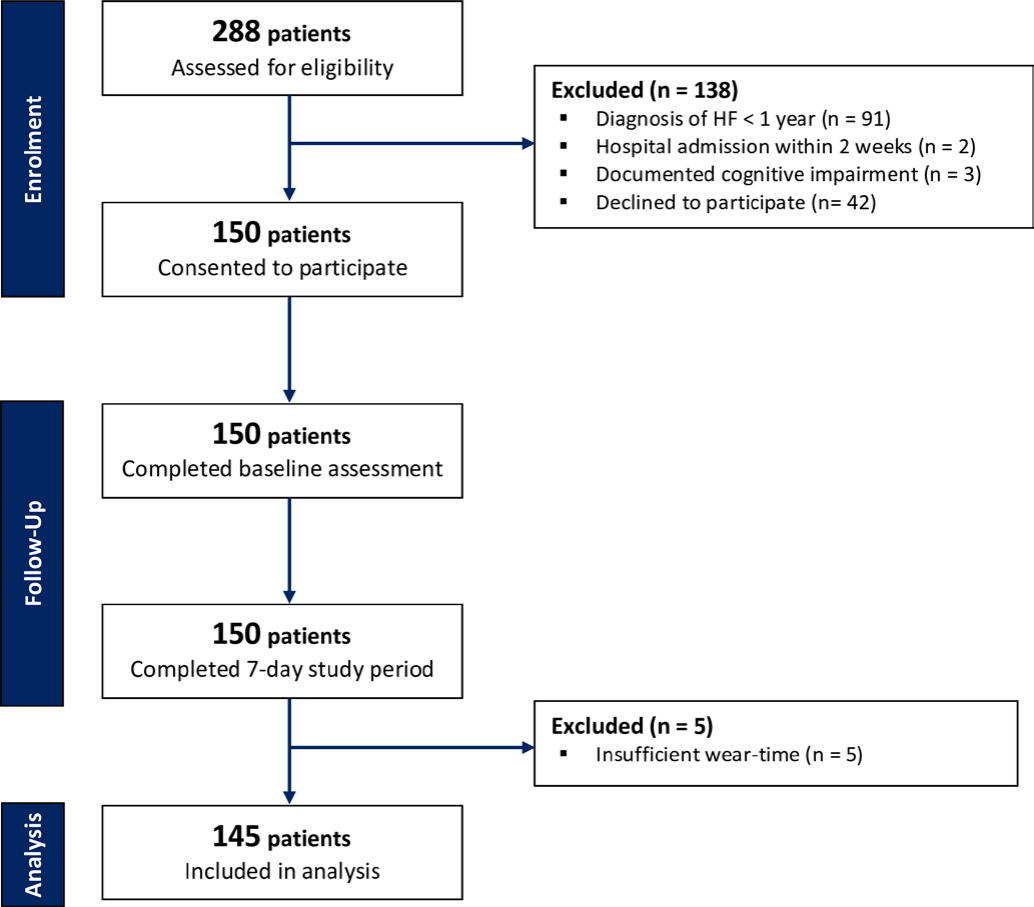
CONSORT diagram of participant flow through the study. CONSORT, Consolidated Standards of Reporting Trials; HF, heart failure.

The flow of study participants is shown in figure 2. A total of 145 participants were included in the final analysis; 5 participants were excluded due to insufficient wear-time. The median age of the study population was 80 (range 65–94 years), 64% were male and 84% were Caucasian. Median NT-proBNP level was 2040 ng/L (IQR 1117–4915), with a normal range of 20–200 ng/L; one-fifth had NYHA class III/IV symptoms; 47% had HFpEF (table 2).

**Table 2:** Demographic characteristics of study participants

Demographics	Study participants* N=145
Age, year—median (range)	80 (65–94)
Gender (male)	93 (64)
Ethnicity	
Caucasian	122 (84)
South Asian	21 (15)
Black	2 (1)
NYHA Class (III/IV)	32 (22)
NT-proBNP, ng/L	2040 (1117–4915)
HF Phenotype	
HFrEF	55 (38)
HFmrEF	22 (15)
HFpEF	68 (47)
Haemoglobin, g/L	128 (115–139)
eGFR, mL/min/1.73 m^2^	45 (33–58)
Frailty (CFS ≥5)	97 (67)
Sleep parameters	
Sleep efficiency, %	81.8 (73.6–86.7)
SPT-window, hour	8.1 (7.0–8.9)
Sleep duration, hour	6.3 (5.4–7.2)
Wake after sleep onset, hour	1.5 (1.1–2.1)
No. of sleep interruptions, n	13 (10–17)
Sleep Regularity Index	45.4 (31.1–56.0)

* Continuous variables reported as median (IQR), categorical variables reported as N (%).

CFS, Clinical Frailty Scale; eGFR, estimated glomerular filtration rate; HF, heart failure; HFmrEF, heart failure with mildly reduced ejection fraction; HFpEF, heart failure with preserved ejection fraction; HFrEF, heart failure with reduced ejection fraction; NT-proBNP, N-terminal pro-B type natriuretic peptide; NYHA, New York Heart Association; SPT, sleep period time.

The median sleep efficiency of the study population was 81.8%. The median SPT window and sleep duration were 8.1 hours/day and 6.3 hours/day, respectively. The number of sleep interruptions experienced throughout the night ranged from 3 to 25 per night (median, 13) (table 2).

Of 145 participants, 42% (n=61) had inefficient sleep (ie, sleep efficiency <80%, averaged across valid nights); they had significantly higher levels of NT-proBNP compared with those with higher sleep efficiency (3141 ng/L vs 1592 ng/L, p=0.044) and were more likely to be South Asian. Patients with inefficient sleep tended to have higher HF symptom burden (NYHA Class III/IV), although this did not reach statistical significance. There was no discernible difference in HF phenotype, frailty status, types or number of comorbidities between the two sleep groups. QoL measures were similar across both groups (table 3). A supplementary analysis comparing various sleep parameters between patients with efficient versus inefficient sleep is presented in online supplemental table S1 (online supplemental material).

**Table 3:** Characteristics of participants with efficient versus inefficient sleep

Demographics	Participants with efficient sleep* (Sleep efficiency ≥80%) N=84	Participants with inefficient sleep* (Sleep efficiency <80%) N=61	P value
Age, year (median, range)	81 (65–94)	78 (66–92)	0.126
Gender (male)	49 (53)	44 (47)	0.087
BMI, kg/m^2^	28 (24–32)	28 (25–32)	0.925
Ethnicity			
Caucasian	77 (63)	45 (37)	0.012
South Asian	6 (29)	15 (71)	
Black	1 (50)	1 (50)	
NT-proBNP, ng/L	1592 (819–4751)	3141 (1330–5646)	0.044
eGFR, mL/min/1.73 m^2^	44.5 (31–57)	45 (34–63)	0.686
HF Phenotype			
HFrEF	30 (55)	25 (45)	0.750
HFmrEF	14 (64)	8 (36)	
HFpEF	40 (59)	28 (41)	
NYHA Class (III/IV)	15 (47)	17 (53)	0.151
Cardiac Devices			
CRT-P	17 (59)	12 (41)	0.933
CRT-D	6 (40)	9 (60)	0.137
Frailty (CFS ≥5)	55 (57)	42 (43)	0.670
Comorbidities†			
Hypertension	37 (55)	30 (45)	0.541
OSA	8 (67)	4 (33)	0.522
AF	54 (61)	34 (39)	0.298
IHD	18 (51)	17 (49)	0.371
COPD	8 (53)	7 (47)	0.703
T2DM	20 (48)	22 (52)	0.108
CKD	26 (50)	26 (50)	0.148
Mental illness‡	4 (100)	0 (0)	0.139
Cancer	23 (79)	6 (21)	0.009
Cerebrovascular disease	8 (80)	2 (20)	0.192
Insomnia	0 (0)	0 (0)	NA
No. of comorbidities, ≥5	14 (48)	15 (52)	0.239
KCCQ-12			
Overall	55.5 (35.7–68.8)	51.6 (34.9–70.3)	0.439
KCCQ-PL	50.0 (33.3–72.9)	41.7 (20.8–58.3)	0.092
KCCQ-SF	60.4 (51.0–75.0)	62.5 (35.4–79.2)	0.578
KCCQ-QoL	62.5 (25.0–75.0)	50.0 (37.5–75.0)	0.872
KCCQ-SL	58.3 (33.3–75.0)	50.0 (33.3–75.0)	0.338
Barthel Index, %	95 (85–100)	90 (75–100)	0.129
Handgrip strength, kg	20 (20–30)	22 (17–28)	0.524
Walk speed, m/sec	0.54 (0.36–0.67)	0.48 (0.35–0.65)	0.373
TUGT, sec	16.1 (11.1–23.9)	19.5 (13.0–24.0)	0.120

* Continuous variables reported as median (IQR), categorical variables reported as N (%).

† Comorbidity data were extracted from participants’ clinical records at the time of recruitment.

‡ Defined as documented diagnosis of anxiety and/or depression.

AF, atrial fibrillation; BMI, body mass index; CFS, Clinical Frailty Scale; CKD, chronic kidney disease; COPD, chronic obstructive pulmonary disease; CRT-D, cardiac resynchronisation therapy-defibrillator; CRT-P, cardiac resynchronisation therapy-pacemaker; eGFR, estimated glomerular filtration rate; HF, heart failure; HFmrEF, heart failure with mildly reduced ejection fraction; HFpEF, heart failure with preserved ejection fraction; HFrEF, heart failure with reduced ejection fraction; IHD, ischaemic heart disease; KCCQ-PL, Kansas City Cardiomyopathy Questionnaire-Physical Limitation; KCCQ-QoL, Kansas City Cardiomyopathy Questionnaire-Quality of Life; KCCQ-SF, Kansas City Cardiomyopathy Questionnaire-Symptom Frequency; KCCQ-SL, Kansas City Cardiomyopathy Questionnaire-Social Limitation; NA, not assessed; NT-proBNP, N-terminal pro-B-type natriuretic peptide; NYHA, New York Heart Association; OSA, obstructive sleep apnoea; T2DM, type 2 diabetes mellitus; TUGT, Timed Up and Go Test.

### Association between sleep parameters and HF severity, functional status and HRQoL

#### Sleep parameters and HF severity

There was no significant association between sleep efficiency and markers of HF severity. Lower SRI (poorer sleep regularity) was associated with higher NYHA class (worse HF symptoms). The association remained significant after accounting for age, gender and number of comorbidities (β=−0.009, p=0.007). There were no significant associations between other sleep parameters and NT-proBNP or LVEF (table 4, online supplemental table S3).

**Table 4:** Linear regression analysis of sleep metrics with HF parameters, functional performance and HRQoL of older people with HF, N=145 (adjusted for age, gender and number of comorbidities)

Variables	Unstandardised coefficient, B (SE)							
	Sleep efficiency	SPT-Window	WASO	Sleep onset	Wake up time	SRI	Sleep duration	No. of sleep interruptions
**HF parameters**								
NT-proBNP	−48.632 (31.560)	213.526 (212.633)	425.610 (331.067)	−427.221 (242.886)	−178.621 (268.900)	4.547 (20.760)	46.264 (233.007)	124.214 (83.702)
LVEF	0.048 (0.089)	0.436 (0.597)	−0.145 (0.933)	−0.508 (0.687)	0.037 (0.755)	0.030 (0.058)	0.591 (0.651)	−0.004 (0.236)
NYHA Class	−0.006 (0.005)	0.004 (0.034)	0.101 (0.053)	0.043 (0.039)	0.056 (0.043)	−0.009* (0.003)	−0.045 (0.037)	0.002 (0.014)
**Functional performance**								
Usual gait speed	0.002 (0.002)	−0.006 (0.011)	−0.039† (0.019)	0.001 (0.013)	−0.008 (0.013)	0.001 (0.001)	0.008 (0.012)	−0.001 (0.004)
Handgrip strength	0.101 (0.058)	0.014 (0.396)	−0.951 (0.612)	0.236 (0.455)	0.326 (0.499)	−0.013 (0.038)	0.482 (0.430)	0.020 (0.156)
Dynamic balance	−0.084 (0.081)	0.409 (0.569)	1.633 (0.942)	−0.511 (0.634)	−0.009 (0.667)	−0.063 (0.050)	−0.193 (0.596)	0.306 (0.205)
ADL	0.271† (0.112)	−1.693† (0.751)	−4.477* (1.131)	1.823† (0.866)	−0.481 (0.964)	0.310* (0.070)	0.174 (0.835)	−0.395 (0.300)
Frailty	−0.017† (0.007)	0.070 (0.047)	0.251* (0.071)	−0.071 (0.054)	0.025 (0.060)	−0.017* (0.004)	−0.040 (0.052)	0.016 (0.019)
**HRQoL**								
KCCQ-Overall	0.276 (0.164)	−0.372 (1.113)	−3.519† (1.712)	0.096 (1.281)	−0.445 (1.405)	0.344* (0.104)	1.278 (1.211)	−0.334 (0.439)
KCCQ-PL	0.347 (0.189)	0.157 (1.283)	−4.170† (1.917)	−0.146 (1.477)	0.135 (1.620)	0.415* (0.120)	2.229 (1.389)	−0.287 (0.507)
KCCQ-SF	0.188 (0.179)	−0.679 (1.202)	−2.721 (1.864)	−0.091 (1.385)	−1.099 (1.516)	0.241† (0.115)	0.522 (1.313)	−0.185 (0.475)
KCCQ-QoL	0.211 (0.208)	0.187 (1.400)	−2.564 (2.174)	−0.481 (1.611)	−0.306 (1.767)	0.219 (0.135)	1.477 (1.524)	−0.234 (0.553)
KCCQ-SL	0.356 (0.205)	−1.155 (1.385)	−4.620† (2.131)	1.101 (1.595)	−0.509 (1.752)	0.500* (0.128)	0.884 (1.514)	−0.627 (0.546)

* Coefficient remains statistically significant after controlling the false discovery rate (α=0.05) using Benjamini-Hochberg procedure (online supplemental table S4).

† Statistically significant at p<0.05 (refer to Table S3 for full results).

ADL, activities of daily living; HF, heart failure; HRQoL, health-related quality of life; KCCQ, Kansas City Cardiomyopathy Questionnaire; KCCQ-PL, Kansas City Cardiomyopathy Questionnaire-Physical Limitation; KCCQ-QoL, Kansas City Cardiomyopathy Questionnaire-Quality of Life; KCCQ-SF, Kansas City Cardiomyopathy Questionnaire-Symptom Frequency; KCCQ-SL, Kansas City Cardiomyopathy Questionnaire-Social Limitation; LVEF, left ventricular ejection fraction; NT-proBNP, N-terminal pro-B type natriuretic peptide; NYHA, New York Heart Association; SPT, sleep period time; SRI, Sleep Regularity Index; WASO, wake after sleep onset.

#### Sleep parameters and functional performance

Lower sleep efficiency was associated with higher CFS (worsening frailty status) (adjusted β=−0.017, p=0.014) and lower BI score (higher dependence on others for ADLs) (adjusted β=0.271, p=0.016) (table 4, online supplemental table S3). Longer WASO was associated with slower gait speed (adjusted β=−0.039, p=0.040), lower BI score (adjusted β=−4.477, p<0.001) and poorer frailty status (adjusted β=0.251, p<0.001). Lower SRI was also associated with lower BI score (adjusted β=0.310, p<0.001) and worse frailty (adjusted β=−0.017, p<0.001).

No associations were observed between sleep parameters and handgrip strength or dynamic balance.

#### Sleep parameters and HRQoL

Sleep efficiency showed no significant association with KCCQ-12 scores. Among other sleep parameters, lower SRI was associated with lower scores across all domains of KCCQ-12, except KCCQ-QoL (table 4, online supplemental table 3). The predictive strength of SRI is strongest for social limitation (adjusted β=0.500, p<0.001), followed by physical limitation (adjusted β=0.415, p<0.001) and frequency of HF symptoms (adjusted β=0.241, p=0.038). Longer night-time awakening (WASO) was associated with poorer HF-related physical function (adjusted β=−4.170, p=0.036) and social limitation (adjusted β=−4.620, p=0.032).

The comprehensive regression results are provided in the Supplementary Material (online supplemental table S2 reports crude estimates prior to covariate adjustment; online supplemental table S3 reports adjusted regression model and online supplemental table S4 presents Benjamini-Hochberg adjusted p values).

## Discussion

In this study, we assessed sleep health in older patients living with HF using wrist actigraphy over a 7-day period. Of the 145 patients studied, we found 42% had inefficient sleep evidenced by actigraphic sleep efficiency. Despite a similar sleep window duration to those with efficient sleep, patients with inefficient sleep experienced remarkably fragmented sleep as recorded by the high number of sleep interruptions during the night and wake after sleep onset (WASO). Consequently, these patients had significantly shorter duration of uninterrupted sleep, with a median of 5.7 hours, to be compared with the 7–8 hours of sleep recommended for older adults.^45^

Notably, median serum NT-proBNP levels were elevated twofold in patients with inefficient sleep compared with those with efficient sleep. As the renal function was similar across the two groups, the higher NT-proBNP levels in patients with inefficient sleep were unlikely to be due to impaired renal clearance and may instead reflect greater prevalence of orthopnoea and paroxysmal nocturnal dyspnoea in the context of more advanced or poorly controlled HF. Alternatively, or additionally, poor sleep might induce activation of the sympathetic nervous system, thus inflicting haemodynamic burden to the heart with the observed effect on NT-proBNP levels. Studies support that sleep restriction^46^ and fragmentation^47 48^ increase heart rate, blood pressure and sympathetic cardiac modulation. Beyond HF severity, higher NT-proBNP levels have also been linked to atrial fibrillation,^49^ sleep-disordered breathing^50^ and dementia.^51^ These observations raise the possibility that low sleep efficiency in HF may reflect higher comorbidity burden, although further studies are required to validate this hypothesis.

In the current study, SRI, a relatively new metric, emerged as an important factor in HF symptom severity. We found that patients with a more irregular sleep/wake pattern experienced more advanced HF symptoms (ie, NYHA class), independent of age, gender and concomitant diseases. These patients had higher ADL dependency and tended to be more frail, with poorer HRQoL outcomes. Prior studies have linked actigraphy-based sleep irregularity with higher risk of incident CVD^52^ and all-cause mortality,^53^ although HF per se was not included in the analyses. Furthermore, poor sleep regularity was found to be a stronger predictor of mortality than sleep duration,^53^ a premise that lends weight to our study findings whereby sleep duration was associated with neither HF severity nor functional performance. This further calls for deeper investigation into SRI as an indicator of cardiovascular health, particularly in older populations with HF. We did not observe an association between LVEF and poor sleep in our population, in keeping with previous studies.^10 13^

Our study also suggests an association between WASO, a surrogate for sleep fragmentation and functional capacity in patients with HF. We found that patients with greater night-time awakening had slower gait speed and were more ADL-dependent with worse frailty status. Expectedly, they expressed lower satisfaction with their overall QoL. Our findings are consistent with the works of Dam and colleagues who reported similar outcomes between WASO and gait speed, although exclusively in older men.^54^ While the interplay between WASO and gait speed in the HF population remains to be fully elucidated, it may involve underlying factors such as insomnia,^10^ excessive daytime sleepiness^55^ and sleep disordered breathing,^56^ which were not explored in the current work. Elsewhere, cognitive–behavioural therapy for insomnia has been shown to produce sustained improvement in Six-Minute Walk Test distance, a prognostic indicator in HF,^57^ alongside improvements in insomnia severity and daytime sleepiness,^58^ in patients with HF and comorbid insomnia. These findings indicate that meaningful treatment of poor sleep can improve physical performance and potentially alter the disease trajectory in this population, although this requires confirmation from independent studies.

Finally, we note with interest the higher prevalence of poor sleep efficiency among South Asian patients compared with Caucasian or Black participants. We might postulate that this may reflect more advanced or poorly controlled HF in South Asian patients; however, neither NYHA class nor NT-proBNP differed between Asian and Caucasian patients. The small sample size further limits a robust statistical analysis and assessment of these findings. Beyond the clinical lens, factors such as acculturation^59^ and socioeconomic background^60^ could also have played a role in the sleep disparity observed within the ethnic minority in our study.

This study has several limitations. First, given the cross-sectional design of the study, we were unable to establish a cause-effect relationship between sleep metrics and clinical parameters, physical function and QoL. Second, participants’ medication usage or sleep hygiene was not studied and their possible confounding effect on sleep pattern cannot be ruled out. However, commonly proscribed sleep hygiene behaviours such as before-bed caffeine consumption, exercise shortly before bedtime and daytime napping do not appear to increase the odds of poor sleep efficiency.^43^ Third, actigraphy measures inactivity as a proxy for sleep.^36^ As such, it is limited in distinguishing true sleep from motionless wakefulness; lying still in bed but awake may be misclassified as sleeping. It has been reported that accelerometers tend to overestimate sleep duration and underestimate WASO, compared with PSG.^61^ While this potentially points toward a poorer sleep health than suggested by the numbers reported in this study, a concurrent assessment using other validated tools (eg, PSQI, ISI or RU SATED^1^ (Regularity, Satisfaction, Alertness, Timing, Efficiency, Duration)) would have served to compare and/or corroborate findings from actigraphy. Fourth, our sample size is limited and based on a single population recruited through convenience sampling, and therefore the observations may not be generalisable. Fifth, as this study was exploratory in nature, *p*-values were interpreted descriptively without multiplicity adjustment; thus, the observed associations should be interpreted with caution and further investigations are recommended to confirm these findings.

## Conclusions

Poor sleep efficiency is common among older patients with HF. 42% of our study participants had inefficient sleep (ie, sleep efficiency <80%); they had significantly higher NT-proBNP levels. Lower sleep efficiency was associated with worse frailty and lower functional independence. Patients with lower sleep regularity experienced more advanced HF symptoms and poorer QoL, in addition to worse frailty status and functional independence. Furthermore, those with greater sleep fragmentation (ie, WASO) demonstrated slower gait speed. Future studies are warranted to investigate mechanisms contributing to poor sleep health in HF populations, with the aim of developing personalised interventions to improve QoL and clinical outcomes in older people with HF.

## Supplementary material

10.1136/bmjopen-2025-111622 online supplemental file 1

## Data Availability

Data are available upon reasonable request.
