# Supplementary material for: Device-assessed sleep health among older patients with heart failure: a cross-sectional study using actigraphy
Source: BMJ Open. 2026 Feb 2;16(2):e111622. doi: 10.1136/bmjopen-2025-111622 (PMC12878371; doi:10.1136/bmjopen-2025-111622)
Supplement: online supplemental file 1 [file bmjopen-16-2-s001.pdf]

Table S1. Comparison of Sleep Parameters between Participants with Efficient *vs* Inefficient Sleep

**Sleep parameter**	**Participants with efficient sleep*<br>(SE ≥ 80%)<br>N = 84**	**Participants with inefficient sleep*<br>(SE < 80%)<br>N = 61**	**P -value**
Sleep Efficiency (%)	85.7 (82.5-88.2)	71 (65.2-76.9)	**<0.001**
Sleep Period Time (SPT) window (hour)	8.0 (6.8-8.7)	8.2 (7.1-9.0)	0.331
Sleep duration (hours)	6.8 (5.9-7.6)	5.7 (4.7-6.3)	**<0.001**
Wake After Sleep Onset (WASO) (hours)	1.1 (0.9-1.4)	2.3 (1.9-3.2)	**<0.001**
Number of Sleep Interruptions	12 (9-16)	14 (11-17)	**0.024**
Sleep Regularity Index (SRI)	47.8 (35.8-60.1)	40.0 (24.4-51.0)	**0.009**
Sleep Onset (HH:MM)	23:14<br>(22:31-00:06)	22:58<br>(22:08–23:42)	0.073
Wake Up Time (HH:MM)	07:21<br>(06:41-07:57)	06:59<br>(06:20-07:54)	0.269

** median (IQR)*

Table S2. Unadjusted linear regression examining associations between sleep metrics with HF parameters, functional performance and HRQoL of older people with HF (N = 145).

Variables	Sleep efficiency		SPT-Window		WASO		Sleep Onset		Wake Up Time		SRI		Sleep Duration		No. of sleep interruptions	
	**B (SE)**	***p*-value**	**B (SE)**	***p*-value**	**B (SE)**	***p*-value**	**B (SE)**	***p*-value**	**B (SE)**	***p*-value**	**B (SE)**	***p*-value**	**B (SE)**	***p*-value**	**B (SE)**	***p*-value**
**HF Parameters**																
**NT-proBNP**	-32.495 (32.451)	0.318	128.238 (220.283)	0.561	232.270 (337.529)	0.492	-321.849 (251.062)	0.202	-194.851 (278.091)	0.485	-0.093 (21.473)	0.997	35.451 (241.901)	0.884	60.563 (86.301)	0.484
**LVEF**	0.075 (0.088)	0.397	0.230 (0.598)	0.701	-0.480 (0.917)	0.601	-0.326 (0.685)	0.635	-0.079 (0.756)	0.917	0.029 (0.058)	0.625	0.523 (0.655)	0.426	-0.066 (0.235)	0.780
**NYHA Class**	-0.004 (0.005)	0.407	-0.009 (0.034)	0.805	0.077 (0.052)	0.142	0.050 (0.039)	0.202	0.046 (0.043)	0.285	-0.009 (0.003)	0.007	-0.050 (0.037)	0.186	-0.002 (0.013)	0.906
**Functional Performance**																
**Usual Gait Speed**	0.002 (0.002)	0.323	0.003 (0.012)	0.816	-0.029 (0.021)	0.174	-0.006 (0.014)	0.688	-0.002 (0.015)	0.891	0.001 (0.001)	0.233	0.015 (0.013)	0.270	0.005 (0.004)	0.312
**Handgrip Strength**	0.001 (0.072)	0.984	0.484 (0.486)	0.321	0.250 (0.748)	0.739	-0.383 (0.558)	0.493	0.337 (0.616)	0.585	-0.018 (0.047)	0.709	0.455 (0.534)	0.396	0.193 (0.191)	0.313
**Dynamic Balance**	-0.063 (0.086)	0.465	0.102 (0.605)	0.866	1.193 (1.000)	0.235	-0.280 (0.672)	0.677	-0.183 (0.707)	0.796	-0.078 (0.053)	0.144	-0.362 (0.633)	0.568	0.128 (0.215)	0.554
**ADL**	0.237 (0.115)	0.041	-1.332 (0.782)	0.091	-3.969 (1.164)	<0.001	1.673 (0.893)	0.063	-0.060 (0.998)	0.952	0.324 (0.072)	<0.001	0.430 (0.866)	0.620	-0.223 (0.309)	0.472
**Frailty**	-0.013 (0.008)	0.086	0.038 (0.053)	0.481	0.201 (0.080)	0.013	-0.052 (0.061)	0.395	-0.005 (0.067)	0.944	-0.019 (0.005)	<0.001	-0.058 (0.058)	0.323	-0.007 (0.021)	0.742
**Health-Related Quality of Life (HRQoL)**																
**KCCQ- Overall**	0.195 (0.168)	0.247	0.179 (1.144)	0.876	-2.513 (1.741)	0.151	-0.298 (1.310)	0.820	-0.033 (1.445)	0.982	0.353 (0.107)	0.001	1.500 (1.249)	0.232	-0.089 (0.448)	0.843

**KCCQ- PL**	0.259 (0.194)	0.184	0.765 (1.318)	0.563	-3.063 (2.007)	0.129	-0.566 (1.510)	0.709	0.605 (1.666)	0.717	0.432 (0.123)	<0.001	2.488 (1.433)	0.085	0.025 (0.517)	0.962
**KCCQ- SF**	0.152 (0.180)	0.401	-0.250 (1.222)	0.838	-2.253 (1.864)	0.229	-0.198 (1.399)	0.888	-0.540 (1.543)	0.727	0.259 (0.117)	0.028	0.852 (1.339)	0.526	-0.002 (0.479)	0.997
**KCCQ- QoL**	0.126 (0.207)	0.543	0.669 (1.403)	0.634	-1.514 (2.148)	0.482	-0.921 (1.605)	0.567	-0.069 (1.773)	0.969	0.214 (0.135)	0.117	1.578 (1.534)	0.305	-0.082 (0.550)	0.882
**KCCQ- SL**	0.245 (0.209)	0.243	-0.470 (1.422)	0.742	-3.221 (2.164)	0.139	0.491 (1.628)	0.764	-0.129 (1.797)	0.943	0.509 (0.132)	<0.001	1.083 (1.558)	0.488	-0.297 (0.557)	0.594

HF, Heart Failure; **HRQoL,** Health-Related Quality of Life; **SPT**, Sleep Period Time; **WASO**, Wake After Sleep Onset; **SRI**, Sleep Regularity Index; **NT-proBNP**, N-terminal pro-B type natriuretic peptide; **LVEF**, Left Ventricular Ejection Fraction; **NYHA,** New York Heart Association; **ADL,** Activities of Daily Living; **KCCQ,** Kansas City Cardiomyopathy Questionnaire; **KCCQ-PL**, Kansas City Cardiomyopathy Questionnaire-Physical Limitation; **KCCQ-SF**, Kansas City Cardiomyopathy Questionnaire-Symptom Frequency; **KCCQ-QoL**, Kansas City Cardiomyopathy Questionnaire-Quality of Life; **KCCQ-SL**, Kansas City Cardiomyopathy Questionnaire-Social Limitation

Table S3. Linear regression analysis of sleep metrics with HF parameters, functional performance and HRQoL of older people with HF, N = 145 (adjusted for age, gender and number of comorbidities)

Variables	Sleep efficiency		SPT-Window		WASO		Sleep Onset		Wake Up Time		SRI		Sleep Duration		No. of sleep interruptions	
	B (SE)	*p*-value	B (SE)	*p*-value	B (SE)	*p*-value	B (SE)	*p*-value	B (SE)	*p*-value	B (SE)	*p*-value	B (SE)	*p*-value	B (SE)	*p*-value
**HF Parameters**																
**NT-proBNP**	-48.632 (31.560)	0.126	213.526 (212.633)	0.317	425.610 (331.067)	0.201	-427.221 (242.886)	0.081	-178.621 (268.900)	0.508	4.547 (20.760)	0.827	46.264 (233.007)	0.843	124.214 (83.702)	0.140
**LVEF**	0.048 (0.089)	0.592	0.436 (0.597)	0.466	-0.145 (0.933)	0.877	-0.508 (0.687)	0.460	0.037 (0.755)	0.961	0.030 (0.058)	0.609	0.591 (0.651)	0.366	-0.004 (0.236)	0.986
**NYHA Class**	-0.006 (0.005)	0.237	0.004 (0.034)	0.918	0.101 (0.053)	0.057	0.043 (0.039)	0.278	0.056 (0.043)	0.194	**-0.009 (0.003)**	**0.007**	-0.045 (0.037)	0.224	0.002 (0.014)	0.871
**Functional Performance**																
**Usual Gait Speed**	0.002 (0.002)	0.160	-0.006 (0.011)	0.572	**-0.039 (0.019)**	**0.040**	0.001 (0.013)	0.945	-0.008 (0.013)	0.552	0.001 (0.001)	0.398	0.008 (0.012)	0.500	-0.001 (0.004)	0.849
**Handgrip Strength**	0.101 (0.058)	0.087	0.014 (0.396)	0.972	-0.951 (0.612)	0.123	0.236 (0.455)	0.605	0.326 (0.499)	0.514	-0.013 (0.038)	0.739	0.482 (0.430)	0.264	0.020 (0.156)	0.899
**Dynamic Balance**	-0.084 (0.081)	0.301	0.409 (0.569)	0.474	1.633 (0.942)	0.085	-0.511 (0.634)	0.421	-0.009 (0.667)	0.990	-0.063 (0.050)	0.214	-0.193 (0.596)	0.747	0.306 (0.205)	0.137
**ADL**	**0.271 (0.112)**	**0.016**	**-1.693 (0.751)**	**0.026**	**-4.477 (1.131)**	**<0.001**	**1.823 (0.866)**	**0.037**	-0.481 (0.964)	0.619	**0.310 (0.070)**	**<0.001**	0.174 (0.835)	0.836	-0.395 (0.300)	0.190
**Frailty**	**-0.017 (0.007)**	**0.014**	0.070 (0.047)	0.141	**0.251 (0.071)**	**<0.001**	-0.071 (0.054)	0.192	0.025 (0.060)	0.677	**-0.017 (0.004)**	**<0.001**	-0.040 (0.052)	0.448	0.016 (0.019)	0.395
**Health-Related Quality of Life (HRQoL)**																
**KCCQ- Overall**	0.276 (0.164)	0.096	-0.372 (1.113)	0.738	**-3.519 (1.712)**	**0.042**	0.096 (1.281)	0.941	-0.445 (1.405)	0.752	**0.344 (0.104)**	**0.001**	1.278 (1.211)	0.293	-0.334 (0.439)	0.449

**KCCQ- PL**	0.347 (0.189)	0.068	0.157 (1.283)	0.903	**-4.170 (1.917)**	**0.036**	-0.146 (1.477)	0.922	0.135 (1.620)	0.934	**0.415 (0.120)**	**<0.001**	2.229 (1.389)	0.111	-0.287 (0.507)	0.572
**KCCQ- SF**	0.188 (0.179)	0.294	-0.679 (1.202)	0.573	-2.721 (1.864)	0.147	-0.091 (1.385)	0.947	-1.099 (1.516)	0.470	**0.241 (0.115)**	**0.038**	0.522 (1.313)	0.692	-0.185 (0.475)	0.697
**KCCQ- QoL**	0.211 (0.208)	0.313	0.187 (1.400)	0.894	-2.564 (2.174)	0.240	-0.481 (1.611)	0.766	-0.306 (1.767)	0.893	0.219 (0.135)	0.106	1.477 (1.524)	0.334	-0.234 (0.553)	0.672
**KCCQ- SL**	0.356 (0.205)	0.085	-1.155 (1.385)	0.406	**-4.620 (2.131)**	**0.032**	1.101 (1.595)	0.491	-0.509 (1.752)	0.772	**0.500 (0.128)**	**<0.001**	0.884 (1.514)	0.560	-0.627 (0.546)	0.253

HF, Heart Failure; **HRQoL,** Health-Related Quality of Life; **SPT**, Sleep Period Time; **WASO**, Wake After Sleep Onset; **SRI**, Sleep Regularity Index; **NT-proBNP**, N-terminal pro-B type natriuretic peptide; **LVEF**, Left Ventricular Ejection Fraction; **NYHA,** New York Heart Association; **ADL,** Activities of Daily Living; **KCCQ,** Kansas City Cardiomyopathy Questionnaire; **KCCQ-PL**, Kansas City Cardiomyopathy Questionnaire-Physical Limitation; **KCCQ-SF**, Kansas City Cardiomyopathy Questionnaire-Symptom Frequency; **KCCQ-QoL**, Kansas City Cardiomyopathy Questionnaire-Quality of Life; **KCCQ-SL**, Kansas City Cardiomyopathy Questionnaire-Social Limitation.

Table S4. Linear regression analysis of sleep metrics with HF parameters, functional performance and HRQoL of older people with HF, with Benjamini-Hochberg adjusted *p*-values†, N = 145

Variables	Sleep efficiency		SPT-Window		WASO		Sleep Onset		Wake Up Time		SRI		Sleep Duration		No. of sleep interruptions	
	Raw *p*-value	BH adj. *p*-value	Raw *p*-value	BH adj. *p*-value	Raw *p*-value	BH adj. *p*-value	Raw *p*-value	BH adj. *p*-value	Raw *p*-value	BH adj. *p*-value	Raw *p*-value	BH adj. *p*-value	Raw *p*-value	BH adj. *p*-value	Raw *p*-value	BH adj. *p*-value
**HF Parameters**																
**NT-proBNP**	0.126	0.234	0.317	0.931	0.201	0.238	0.081	0.527	0.508	0.990	0.827	0.827	0.843	0.843	0.140	0.822
**LVEF**	0.592	0.592	0.466	0.931	0.877	0.877	0.460	0.912	0.961	0.990	0.609	0.720	0.366	0.793	0.986	0.986
**NYHA Class**	0.237	0.339	0.918	0.972	0.057	0.106	0.278	0.904	0.194	0.990	0.007	**0.015**	0.224	0.793	0.871	0.974
**Functional Performance**																
**Usual Gait Speed**	0.160	0.260	0.572	0.931	0.040	0.091	0.945	0.947	0.552	0.990	0.398	0.517	0.500	0.809	0.849	0.974
**Handgrip Strength**	0.087	0.208	0.972	0.972	0.123	0.178	0.605	0.947	0.514	0.990	0.739	0.801	0.264	0.793	0.899	0.974
**Dynamic Balance**	0.301	0.339	0.474	0.931	0.085	0.138	0.421	0.912	0.990	0.990	0.214	0.309	0.747	0.843	0.137	0.822
**ADL**	0.016	0.104	0.026	0.338	<0.001	**0.013**	0.037	0.481	0.619	0.990	<0.001	**0.013**	0.836	0.843	0.190	0.822
**Frailty**	0.014	0.104	0.141	0.917	<0.001	**0.013**	0.192	0.832	0.677	0.990	<0.001	**0.013**	0.448	0.809	0.395	0.973
**Health-Related Quality of Life (HRQoL)**																
**KCCQ- Overall**	0.096	0.208	0.738	0.972	0.042	0.091	0.941	0.947	0.752	0.990	0.001	**0.013**	0.293	0.793	0.449	0.973

**KCCQ- PL**	0.068	0.208	0.903	0.972	0.036	0.091	0.922	0.947	0.934	0.990	<0.001	**0.013**	0.111	0.793	0.572	0.974
**KCCQ- SF**	0.294	0.339	0.573	0.931	0.147	0.191	0.947	0.947	0.470	0.990	0.038	0.071	0.692	0.843	0.697	0.974
**KCCQ- QoL**	0.313	0.339	0.894	0.972	0.240	0.260	0.766	0.947	0.893	0.990	0.106	0.172	0.334	0.793	0.672	0.974
**KCCQ- SL**	0.085	0.208	0.406	0.931	0.032	0.091	0.491	0.912	0.772	0.990	<0.001	**0.013**	0.560	0.809	0.253	0.822

†controlled for age, gender and number of comorbidities

BH adj. p-value, Benjamini- Hochberg adjusted p-value**; HF,** Heart Failure; **HRQoL,** Health-Related Quality of Life; **SPT**, Sleep Period Time; **WASO**, Wake After Sleep Onset; **SRI**, Sleep Regularity Index; **NT-proBNP**, N-terminal pro-B type natriuretic peptide; **LVEF**, Left Ventricular Ejection Fraction; **NYHA,** New York Heart Association; **ADL,** Activities of Daily Living; **KCCQ,** Kansas City Cardiomyopathy Questionnaire; **KCCQ-PL**, Kansas City Cardiomyopathy Questionnaire-Physical Limitation; **KCCQ-SF**, Kansas City Cardiomyopathy Questionnaire-Symptom Frequency; **KCCQ-QoL**, Kansas City Cardiomyopathy Questionnaire-Quality of Life; **KCCQ-SL**, Kansas City Cardiomyopathy Questionnaire-Social Limitation
