# Supplementary material for: Device-assessed sleep health among older patients with heart failure: a cross-sectional study using actigraphy
Source: BMJ Open. 2026 Feb 2;16(2):e111622. doi: 10.1136/bmjopen-2025-111622 (PMC12878371; doi:10.1136/bmjopen-2025-111622)
Supplement: Supplementary file 2 [file bmjopen-2025-111622.reviewer_comments.pdf]

PEER REVIEW HISTORY

BMJ Open publishes all reviews undertaken for accepted manuscripts. Reviewers are asked to complete a checklist review form and are provided with free text boxes to elaborate on their assessment. These free text comments are reproduced below.

ARTICLE DETAILS

Title (Provisional)

Device-assessed sleep health among older patients with heart failure: A cross-sectional study using actigraphy

Authors

Krishnan, Sunanthiny; Taylor, Shelley; Edwardson, Charlotte; Rowlands, Alex; Squire, Iain; Sze, Shirley

---

VERSION 1 - REVIEW

**Reviewer**	**1**
**Name**	**Lavie, Carl**
**Affiliation**	**The University of Queensland School of Medicine**
**Date**	**23-Oct-2025**
**COI**	**None**

Although there are no hard clinical end points and there are other papers on Sleep in HF, there is enough unique and alot of data points to justify publication. They could consider a very recent major State of the Art on Sleep and CVD (Jaspan VN et al. Curr Athero Reports 2024;26:249-262.) Also , unless I missed it , did they assess cardiorespiratory fitness or physical activity /steps per day and cadence ?

**Reviewer**	**2**
**Name**	**Pacheco, André**
**Affiliation**	**Oslo University Hospital, Department of Research and Innovation, Division of Mental Health and Addiction**
**Date**	**25-Oct-2025**
**COI**	**None**

Reviewer report for Krishnan et al

The authors have composed an interesting paper on an important topic. Little is known about sleep efficiency and sleep regularity in patients with heart failure, so this paper adds an important contribution to the field. Their cross-sectional study provides a first exploratory

glance at older patients with heart failure, key clinical characteristics and their link to inefficient or irregular sleep. However, before publication, some points require consideration and revision.

Overall, I suggest you change to more "people-inclusive" language related to these older adults with low sleep efficiency. Meaning that in Table 1, replace "Good sleepers" with what you have measured – "Efficient sleep". Similarly, replace "Poor sleepers" with "Inefficient sleep". Including the set threshold of sleep efficiency, as you have done, is wise within that context. An even more inclusive option is measurement-aligned wording (e.g., 'participants with low/high actigraphy-derived sleep efficiency [threshold: <80%/≥80%]') rather than 'poor/good sleepers' or 'sleep quality.' Both options clarify the construct and reduces ambiguity with subjective sleep quality.

Abstract

1. The limited word count limits the possibility to link the different aspects in your introductory part, but I suggest you try to bridge the passage between heart failure, poor sleep and health outcomes.

2. "Poor sleep quality"; please revise to "Inefficient sleep", and then describe the threshold you used for this.

3. Conclusion in abstract – please revise so that you describe how older adults with HF with inefficient sleep had significantly higher NTproBNP-levels, etcetera.

Introduction

4. This part is theoretically solid but lacks a deeper (albeit brief) introduction to sleep health, a term which you end up using later in the paper. Briefly introducing the concept, successfully introduced by Buysse (2014, PMID: 24470692), and its relevance, would align well with your manuscript's title.

5. I suggest you refine your definition of fragmented or inefficient sleep, to reflect just that. There are a plethora of different ways to assess sleep, which yield quite distinct estimates when it comes to e.g., sleep duration and efficiency. Sleep quality is an exclusively subjective phenomenon – although unlikely, a person could theoretically have an abysmal sleep efficiency, spend lots of time in their bed, but still feel they have good sleep quality. In this sense, instruments such as PSQI, although subject to recall bias, gauge discomfort related to sleep, and capture a more complex phenomenon. Even sleep diaries with simple Likert-type scales related to sleep quality (less susceptible to recall bias), likely capture a different phenomenon compared with actigraphically measured sleep. Therefore, to be more precise, I suggest you revise the term "poor sleep" to "inefficient sleep" throughout the manuscript. Similarly "good sleepers" and "poor sleepers" should be revised to "Efficient sleep" and "Inefficient sleep", characterising the participants not with their sleep, but describing the group they belong to. Although a minor point, this can serve to be more people-inclusive and highlights the plasticity of sleep, which can be improved through cognitive-behavioural interventions.

6. In the introduction or discussion section, please add newer references in addition to REFs 21 and 22, that specifically consider the limitations of actigraphy when investigating disordered sleep, e.g. PMID: 29991438, which in general indicates overestimation of TST compared with PSG, and tends to underestimate WASO vs PSG. I suggest acknowledging this potential limitation in your discussion.

Methods

7. Overall, this part reads well, but I suggest you add the reference by AJK Phillips et al (28607474) introducing and defining the sleep regularity index.

8. You mention taking a full blood count and assessing renal function. Yet, you do not report this. Please state why, and if available and possible, make this data available either in the Supplementary material divided by those with high sleep efficiency (>80%) and low sleep efficiency (<80%). E.g., reporting renal function is important for NTproBNP clearance. It could perhaps be so that there is a difference in renal function between the groups, which could explain the apparent difference? (If not, this would bolster the notion that some other mechanisms might be at play, e.g., sleep parameters influencing this variable).

9. Did you collect data on medication usage? If so, please report this in the Supplement, as different medications used by patients with HF, e.g., diuretics might impact sleep through increased propensity for nocturia.

10. You describe the sampling frequency in your actigraphic recording. Please also describe your epoch length. Please also describe the performance of the GENEactiv triaxial accelerometer to detect wake/sleep compared with PSG (referencing the unit's validation paper when compared with PSG, if there is such a paper).

11. Reproducibility for sleep regularity requires mentioning a few aspects. For your sleep regularity calculation using GGIR v.3.0-9, please report whether you are using SRI1 or SRI2. Please also report window size, and use the novel RIRI checklist to report further metrics related to assessment of sleep regularity (Czeisler et al., 2025, 10.1093/sleep/zsaf299 – the checklist is found in its entirety in the supplement of that article, page 28-29), that have not been mentioned in your current manuscript, which among other things include window length, accelerometer 2-stage sleep/wake detection performance vs PSG, mathematical formulae (for SRI, this would be the Phillips et al., 2017 paper).

Results

12. I suggest you provide the reader a flowchart with the number of people who were assessed for eligibility to partake in the study, and reasons for exclusion, to get a grasp of the selection process in this study and the representativity of the present sample.

13. Table 3, how do you classify comorbidities, via ICD-10 codes from their hospital medical records? And how did you define "mental illness"?

14. Given multiple related tests, please either note that p-values are interpreted descriptively without multiplicity adjustment or provide Benjamini-Hochberg or FDR-adjusted p-values in a supplementary table.

Discussion

15. Similarly, as mentioned earlier, revise relevant aspects, e.g., “poor sleepers” to “those with inefficient sleep”, or “low sleep efficiency”.

16. You may want to consider tempering that given the exploratory nature of your study, and various statistical tests without correction for multiple-testing, further longitudinal studies should assess whether these results can be reproduced.

17. Your observation related to NTproBNP levels is interesting. You can consider introducing the broader relevance of this association, as this marker is also associated with an increased risk of incident dementia (PMID: 31865797). This aspect is also crucial related to the link between NTproBNP levels and atrial fibrillation, and the haemodynamic effects, and potentially NTproBNP-elevating effects of obstructive sleep apnoea (PMID: 16598442).

18. You should also consider the role unmeasured sleep-disordered breathing (affecting around 38% of patients with HF, PMID: 38722388) may play in your sample with regard to fragmented sleep besides orthopnoea and dyspnoea.

19. Another confounder is insomnia, which might be quite common in this group. Moreover, given the propensity of patients with insomnia to spend excessive time in bed – lying still – the actigraph might not capture actual wake periods. Please include this in your limitations section.

20. Another limitations you can consider, is actually the absence of a validated measure of sleep quality in this cohort., e.g., the PSQI (Buysse et al., 1989), or more recent methods to assess (multidimensional) sleep health which include satisfaction with sleep and sleep quality, e.g., the RU-SATED (v.4.0), which considers regularity, satisfaction with sleep, alertness, sleep timing, excessive daytime sleepiness, efficiency of sleep and sleep duration.

21. In patients with HF and chronic insomnia (characterised by low sleep efficiency and daytime impairment), group cognitive-behavioural therapy for insomnia can ameliorate insomnia symptoms and improve physical performance (6 min walking distance test). So, while larger studies might indeed be needed to further elucidate links between WASO and gait speed, well-designed interventional studies can also serve to illustrate core mechanisms. You may want to consider adding that inefficient sleep, and poor sleep quality in patients with HF with insomnia, can be meaningfully treated (Redeker et al., 2022, PMID: 34657160). Crucially, this kind of treatment might reduce functional impairment associated with poor sleep in patients with HF.

**Reviewer**	**3**
**Name**	**Dack, Kyle**

**Affiliation**	**University of Bristol, MRC Integrative Epidemiology Unit**
**Date**	**12-Nov-2025**
**COI**	**None**

Statistical review summary:

The methodological reporting is clear, and suitable statistical methods are applied. My sole major concern is multiple testing. Given that there are a very high number of significance tests performed, some significant findings are likely to be due to chance alone (I counted over 100 p-values). This limits how reliable the results can be considered, and I have given suggestions below for ways this could be addressed, either by streamlining the analysis or applying a statistical correction.

Abstract:

1. The timeframe of the study is not clear from the abstract, and seeking more information in the methods did not fully clarify it, particularly the timing of the various outcomes. (a) the test outcomes – in the methods it sounds like these were evaluated on the "baseline" day, but from the text it is not clear if this is day 1 of wrist tracking, or day 0? (b) sleep outcomes – each individual is tracked for 7 days so will have multiple sleep outcomes, are these averaged, or is a certain day used?

I suggest briefly clarifying in the abstract methods and main text methods, because it impacts how the results can be interpreted.

2. "Regression analysis was used to examine associations between sleep outcomes and other variables" this would be clearer if made more specific e.g. "… between sleep outcomes and the previously stated tests and assessments".

3. "42% had poor sleep quality" – on average, for a specific day, or for at least 1 day of the study period?

Methods:

4. As mentioned in the abstract comments I suggest either adding an "overview" subheading to "data collection" which summarises the timing of baseline assessments, actigraphy, and sleep parameters, or add details to the existing subheadings to ensure readers can easily follow how these all link.

5. For each data collection item I suggest adding the units which it was coded in, to ensure full statistical transparency and to ensure readers interpret the results accurately. For example, a sentence at the end of each to state that it was recorded in x units, or had a scale of x to y. This was reported for ADL and frailty, but not others.

6. Table 1. These parameters all describe a single day, but the participants were followed for up to 7 days. I imagine the mean value was used, but there are alternative, so this should be directly stated in the methods.

7. Modelling strategy: To align with STROBE I recommend adding more details – (a) that both unadjusted and adjusted regression was performed, (b) listing all 8 sleep outcomes, and (c) listing all exposures. This will help to prepare readers for the results, ensuring they understand accurately, and do not expect anything different. For example, it was stated that sleep efficiency was the primary proxy, wo readers could expect that only this will be modelled.

8. Sleep efficiency: This is part analysed as a binary variable (good vs poor sleeper) and part as a continuous (in the main model). I suggest making this consistent, or explaining the rationale for changing it.

9. Multiple testing: there are over 100 p-values in the results, which greatly increases the likelihood of a significant finding due to chance and undermines the confidence readers can have in the results.

-The authors could account for multiple testing by applying a p-value correction, and/or streamlining the analysis. For example:

-Setting a primary outcome. Sleep efficiency was described as a primary proxy but this hierarchy is not applied to the methods where all outcomes are presented equally.

-Table 3 and the unadjusted results in Table 5 are conceptually making the same comparisons. Unadjusted results also have limited interpretability due to confounding. Could the unadjusted results be kept as an appendix?

-Table 4 reports statistics which are outside of the aims of the study – it compares SE categories against the other sleep outcomes. I am unsure what the reader should take from this – is it that the outcomes tend to align and therefore SE is valid as a primary proxy? I am not sure of the purpose of the p-values and suggest the authors consider if they are needed, either integrating it with the aims more or perhaps moving it to an appendix.

-Streamlining the exposures if there are some with poor variance and therefore weak statistical power.

Results

10. The results are all very clearly reported, including the tables. All information necessary is reported, and there is a good level of detail in the text. My only minor comments relate to table 5. “model 1 and 2” would be much clearer just stating “unadjusted” and “adjusted”. Or even better by splitting them into two tables, for the reasons mentioned above. The N of the models should be reported to align with STROBE.

Discussion

11. The interpretation is justified by the findings, and has a good balance between caution and inference. In the limitations I suggest evaluating the risk of confounding bias – the authors adjusted for three confounders, are there any other plausible factors? If not, this is worth also stating, as a strength.

VERSION 1 - AUTHOR RESPONSE

No.	Reviewer's Comments	Authors' Response
**Reviewer #1**<br>**(Dr. Carl Lavie, The University of Queensland School of Medicine)**		
1.	Although there are no hard clinical end points and there are other papers on Sleep in HF, there is enough unique and a lot of data points to justify publication. They could consider a very recent major State of the Art on Sleep and CVD (Jaspan VN et al. Curr Athero Reports 2024;26:249-262.) Also, unless I missed it , did they assess cardiorespiratory fitness or physical activity /steps per day and cadence ?	Thank you for the positive feedback and the suggestion of the review paper. We value the relevance of the article and have incorporated it in the manuscript.<br><br>We did not perform cardiopulmonary exercise testing on our study participants as it was not part of our original aim for the present work.<br><br>We did undertake an in-depth analysis of their physical activity profile from the accelerometers. However, given the large volume and complexity of these data, we chose not to include those results here in order to maintain a clear focus on the primary objective of this manuscript, namely evaluation of participants' sleep pattern.

Reviewer #2
(Dr. André Pacheco, Oslo University Hospital, University of Oslo Faculty of Medicine)

1.	Overall, I suggest you change to more "people-inclusive" language related to these older adults with low sleep efficiency. Meaning that in Table 1, replace "Good sleepers" with what you have measured – "Efficient sleep". Similarly, replace "Poor sleepers" with "Inefficient sleep". Including the set threshold of sleep efficiency, as you have done, is wise within that context. An even more inclusive option is measurement-aligned wording (e.g., 'participants with low/high actigraphy- derived sleep efficiency [threshold: <80%/≥80%]') rather than 'poor/good sleepers' or 'sleep quality.' Both options clarify the construct and reduces ambiguity with subjective sleep quality.	Thank you for bringing this to our attention.<br><br>We acknowledge the importance of people-inclusive language in research and in scientific writing.<br><br>We have now removed the terms "*good/ poor sleepers*" in the manuscript and replaced with "***participants with efficient/inefficient sleep***", reflecting the outcome measured.
2.	*Abstract*<br>The limited word count limits the possibility to link the different aspects in your introductory part, but I suggest you try to bridge the passage between heart failure, poor sleep and health outcomes	The **Abstract** has been revised to reflect reviewer's feedback
3.	*Abstract*<br>"Poor sleep quality"; please revise to "Inefficient sleep", and then describe the threshold you used for this.	The term '*poor sleep quality*' has now been replaced with '***inefficient sleep'*** as suggested.
4.	*Conclusion in Abstract*<br>Please revise so that you describe how older adults with HF with inefficient sleep had significantly higher NTproBNP-levels, etcetera.	The text has now been revised to reflect more inclusive language as recommended.
5.	*Introduction*<br>This part is theoretically solid but lacks a deeper (albeit brief) introduction to sleep health, a term which you end up using later in the paper. Briefly introducing the concept, successfully introduced by Buysse (2014, PMID: 24470692), and its relevance, would align well with your manuscript's title.	We have now included the definition of sleep health (Buysse 2014) in the **Introduction** to ensure clarity of the concept.

6.	*Introduction*<br>I suggest you refine your definition of fragmented or inefficient sleep, to reflect just that. There are a plethora of different ways to assess sleep, which yield quite distinct estimates when it comes to e.g., sleep duration and efficiency. **Sleep quality is an exclusively subjective phenomenon** – although unlikely, a person could theoretically have an abysmal sleep efficiency, spend lots of time in their bed, but still feel they have good sleep quality. In this sense, instruments such as PSQI, although subject to recall bias, gauge discomfort related to sleep, and capture a more complex phenomenon. Even sleep diaries with simple Likert-type scales related to sleep quality (less susceptible to recall bias), likely capture a different phenomenon compared with actigraphically measured sleep.<br><br>Therefore, to be more precise, I suggest you revise the term "poor sleep" to "inefficient sleep" throughout the manuscript. Similarly "good sleepers" and "poor sleepers" should be revised to "Efficient sleep" and "Inefficient sleep", characterising the participants not with their sleep, but describing the group they belong to. Although a minor point, this can serve to be more people-inclusive and highlights the plasticity of sleep, which can be improved through cognitive-behavioural interventions.	We acknowledge the limitation of actigraphy in that it does not quite capture the subjective satisfaction of sleep which is what is defined as *sleep quality* by Buysse (2014).<br><br>In order to avoid any ambiguity, as recommended by the reviewer, we have revised the manuscript and have amended all reference to *'poor sleep quality/ poor sleepers'* to ***'inefficient sleep/ participants with inefficient sleep'***, as contextually appropriate.
7.	*Introduction*<br>In the introduction or discussion section, please add newer references in addition to REFs 21 and 22, that specifically consider the limitations of actigraphy when investigating disordered sleep, e.g. PMID:<br>29991438, which in general indicates	We recognise the inherent limitations of actigraphy in accurately characterising sleep patterns, particularly the potential misclassification of 'still wakefulness' as 'sleep', which may result in overestimation of TST and underestimation of WASO.

	overestimation of TST compared with PSG, and tends to underestimate WASO vs PSG. I suggest acknowledging this potential limitation in your discussion.	We have now clarified this under **Limitation,** integrating our response to Comment No. 20, with supporting reference.
8.	*Methods*<br>Overall, this part reads well, but I suggest you add the reference by AJK Phillips et al (28607474) introducing and defining the sleep regularity index.	Thank you.<br>We have now added the reference for sleep regularity index in **Table 1** within the **Methods** section.
9.	*Methods*<br>You mention taking a full blood count and assessing renal function. Yet, you do not report this. Please state why, and if available and possible, make this data available either in the Supplementary material divided by those with high sleep efficiency (>80%) and low sleep efficiency (<80%). E.g., reporting renal function is important for NTproBNP clearance. It could perhaps be so that there is a difference in renal function between the groups, which could explain the apparent difference? (If not, this would bolster the notion that some other mechanisms might be at play, e.g., sleep parameters influencing this variable).	We have now provided the estimated glomerular filtration rate (eGFR) of participants in **Table 3,** comparing participants with efficient *vs* inefficient sleep. We have linked it to the **Discussion** on the observed difference in NT-proBNP in the study.<br><br>Additionally, we have now reported the median eGFR and haemoglobin levels of the study population in **Table 2,** *Demographic Characteristics of Study Participants*
10.	*Methods*<br>Did you collect data on medication usage? If so, please report this in the Supplement, as different medications used by patients with HF, e.g., diuretics might impact sleep through increased propensity for nocturia.	We did not collect data on participants' medication use during the study period. We agree that data on medication use would have offered additional insight into the sleep health of our study population.<br><br>We note this as a **Limitation** of the present work.
11.	*Methods*<br>You describe the sampling frequency in your actigraphic recording. Please also describe your epoch length. Please also describe the performance of the GENEactiv triaxial accelerometer to detect wake/sleep compared with PSG (referencing the unit's validation paper	Raw data from the accelerometers were converted into 5 second epochs.<br><br>Relative to polysomnography, the accuracy and C-statistic for SPT-window classification by GENEActiv accelerometer has been reported to be

	when compared with PSG, if there is such a paper).	87% and 0.86, respectively. The average sensitivity to detect sleep as part of the SPT-window is above 91%[1].<br><br>We have now included this information within the **Accelerometry Data Processing** section, with additional details on GGIR processing.
12.	*Methods*<br>Reproducibility for sleep regularity requires mentioning a few aspects. For your sleep regularity calculation using GGIR v.3.0-9, please report whether you are using SRI1 or SRI2. Please also report window size, and use the novel RIRI checklist to report further metrics related to assessment of sleep regularity (Czeisler et al., 2025, 10.1093/sleep/zsaf299 – the checklist is found in its entirety in the supplement of that article, page 28-29), that have not been mentioned in your current manuscript, which among other things include window length, accelerometer 2-stage sleep/wake detection performance vs PSG, mathematical formulae (for SRI, this would be the Phillips et al., 2017 paper).	Thank you for drawing our attention to the RIRI statement.<br><br>We used the SRI1 outcome in GGIR.<br>We have now provided all the relevant information on SRI calculation in accordance with the checklist, within the **Methods** and **Results** sections.
13.	*Results*<br>I suggest you provide the reader a flowchart with the number of people who were assessed for eligibility to partake in the study, and reasons for exclusion, to get a grasp of the selection process in this study and the representativity of the present sample.	We have now included a CONSORT diagram detailing participant flow through the study, under the Results section **(Figure 2)**
14.	*Results*<br>Table 3, how do you classify comorbidities, via ICD-10 codes from their hospital medical records? And how did you define "mental illness"?	We retrieved information on comorbidities directly from participants' clinical notes at the time of recruitment, without the use of ICD-10 coding. We have now provided this information as a footnote under **Table 3**.<br><br>For '*mental illness*', we used the **DSM-5-TR**[2] as a guide to categorise the diagnoses. For this study, any health condition listed

		in DSM-5-TR was considered a *'mental illness'*. In our cohort, this encompassed documented diagnosis of ***depression*** and ***anxiety***.<br><br>We have now provided this information as a footnote under **Table 3** for greater transparency.<br><br>We intentionally presented Obstructive Sleep Apnoea (OSA) on its own in the table (although it falls under DSM-5 Sleep-Wake Disorders), given its known high prevalence in patients with HF.
15.	*Results*<br>Given multiple related tests, please either note that p-values are interpreted descriptively without multiplicity adjustment or provide Benjamini-Hochberg or FDR-adjusted p-values in a supplementary table.	Thank you for the suggestion. We acknowledge the inflated risk of Type I error with the multiple testing in the study.<br><br>We have now stated explicitly (under **Statistical Analysis**) that *p*-values are interpreted descriptively without multiplicity adjustment.<br><br>In addition, we have presented Benjamini-Hochberg adjusted *p*-values controlling the false discovery rate (α = 0.05) in the Supplementary material (**Table S4**). Associations that remained significant after BH correction are indicated with asterisks (**) in **Table 4**† with accompanying footnote.<br><br>†We were unable to present the *p*-values in Table 4 (in the main manuscript), due to the tables formatting requirement of the journal (our previous regression table was deemed too large; maximum columns allowed = 9). Instead, we have indicated unstandardised coefficients that were statistically significant with an asterisk (*) in Table 4 and signposted readers to Supplementary **Table S3** for the full regression results.
16.	*Discussion*<br>Similarly, as mentioned earlier, revise relevant aspects, e.g., "poor sleepers"	We have removed the terms "*good/ poor sleepers*" in the manuscript and replaced

	to “those with inefficient sleep”, or “low sleep efficiency”.	with “***participants with efficient/ inefficient sleep***”
17.	*Discussion*<br>You may want to consider tempering that given the exploratory nature of your study, and various statistical tests without correction for multiple-testing, further longitudinal studies should assess whether these results can be reproduced.	We have revisited the manuscript to ensure we are not making bold conclusions on the results, in keeping with the limitations of our study.<br><br>We have also highlighted this in the **Limitation** section.
18.	*Discussion*<br>Your observation related to NTproBNP levels is interesting. You can consider introducing the broader relevance of this association, as this marker is also associated with an increased risk of incident dementia (PMID: 31865797). This aspect is also crucial related to the link between NTproBNP levels and atrial fibrillation, and the haemodynamic effects, and potentially NTproBNP-elevating effects of obstructive sleep apnoea (PMID: 16598442).	Thank you for the suggestion.<br>We have expanded the **Discussion**, incorporating the point suggested.
19.	*Discussion*<br>You should also consider the role unmeasured sleep-disordered breathing (affecting around 38% of patients with HF, PMID: 38722388) may play in your sample with regard to fragmented sleep besides orthopnoea and dyspnoea.	We appreciate this feedback.<br>We have acknowledged this in the following sentence, in the **Discussion**:<br><br>*“Whilst the interplay between WASO and gait speed in HF population remains to be fully elucidated, it may involve underlying factors such as insomnia,[10] excessive daytime sleepiness[55] and sleep disordered breathing,[56] which were not explored in the current work”*
20.	*Discussion*<br>Another confounder is insomnia, which might be quite common in this group. Moreover, given the propensity of patients with insomnia to spend excessive time in bed – lying still – the actigraph might not capture actual wake periods. Please include this in your limitations section.	Thank you for highlighting this point.<br><br>We acknowledge that insomnia is highly prevalent in HF population & in older adults. However, in our study cohort, none of the patients had documented comorbid insomnia. On this background, we did not initially consider insomnia as a confounder. We have now provided this

		information in **Table 3: Comorbidities,** for greater transparency.<br><br>At the same time, we do not discount the possibility of *undiagnosed* insomnia in this population[3] and in the absence of concurrent assessment (using validated tools), we agree that the possible confounding effect of insomnia cannot be ruled out. We have now acknowledged the role of unmeasured insomnia, in the **Discussion**.<br><br>We have also addressed the limitation of actigraphy in distinguishing true sleep from motionless wakefulness (potentially overestimating sleep duration and underestimating wake after sleep onset (WASO)) under **Limitation**, incorporating response to Comment No.7.
21.	*Discussion*<br>Another limitations you can consider, is actually the absence of a validated measure of sleep quality in this cohort., e.g., the PSQI (Buysse et al., 1989), or more recent methods to assess (multidimensional) sleep health which include satisfaction with sleep and sleep quality, e.g., the RU-SATED (v.4.0), which considers regularity, satisfaction with sleep, alertness, sleep timing, excessive daytime sleepiness, efficiency of sleep and sleep duration.	We agree that a concurrent assessment using a validated tool would have been useful in comparing/corroborating findings from actigraphy.<br><br>We note this as a **Limitation** of this present work.
22.	*Discussion*<br>In patients with HF and chronic insomnia (characterised by low sleep efficiency and daytime impairment), group cognitive-behavioural therapy for insomnia can ameliorate insomnia symptoms and improve physical performance (6 min walking distance test). So, while larger studies might indeed be needed to further elucidate links between WASO and gait speed, well-designed interventional studies can also serve to illustrate core mechanisms. You may want to	Thank you for offering this insight.<br><br>We have now incorporated the point raised about CBT-I and functional performance in our **Discussion.**

	consider adding that inefficient sleep, and poor sleep quality in patients with HF with insomnia, can be meaningfully treated (Redeker et al., 2022, PMID: 34657160). Crucially, this kind of treatment might reduce functional impairment associated with poor sleep in patients with HF.	
**Reviewer #3<br>(Dr. Kyle Dack, University of Bristol)**		
1.	*Statistical review summary:*<br>The methodological reporting is clear, and suitable statistical methods are applied. My sole major concern is multiple testing. Given that there are a very high number of significance tests performed, some significant findings are likely to be due to chance alone (I counted over 100 p-values). This limits how reliable the results can be considered, and I have given suggestions below for ways this could be addressed, either by streamlining the analysis or applying a statistical correction.	Thank you for the feedback.<br><br>We acknowledge the reviewer's concern regarding multiple testing and have taken onboard recommendations (below) to improve the scientific rigour of this paper.
2.	*Abstract*<br>The timeframe of the study is not clear from the abstract, and seeking more information in the methods did not fully clarify it, particularly the timing of the various outcomes. (a) the test outcomes – in the methods it sounds like these were evaluated on the "baseline" day, but from the text it is not clear if this is day 1 of wrist tracking, or day 0? (b) sleep outcomes – each individual is tracked for 7 days so will have multiple sleep outcomes, are these averaged, or is a certain day used?<br>I suggest briefly clarifying in the abstract methods and main text methods, because it impacts how the results can be interpreted.	(a) The baseline assessments were conducted on Day 0. Participants were fitted with the accelerometer after completing the baseline assessments, with the device configured to start recording the following day, Day 1.<br><br>(b) The sleep outcomes were averaged across all valid nights<br><br>We have now added this information in the **Abstract** and **Methods** (*Sleep Parameters*) section of the main text.

3.	*Abstract*<br>"Regression analysis was used to examine associations between sleep outcomes and other variables" this would be clearer if made more specific e.g. "… between sleep outcomes and the previously stated tests and assessments".	Thank you for the suggestion.<br>We have now rephrased the sentence for better clarity.

4.	*Abstract*<br>"42% had poor sleep quality" – on average, for a specific day, or for at least 1 day of the study period?	Sleep efficiency* was calculated as an average of valid nights within the 7-day study period.<br><br>We have now revised the **Methods** section of the Abstract (as well as the main text) to reflect this information.<br><br>*In the revised manuscript, the term '*poor sleep quality*' has been replaced with ***'inefficient sleep'***, to better align with the construct being measured. Accordingly, the terms *'good sleepers/ poor sleepers'* have been replaced with ***'participants with efficient/ inefficient sleep'***.
5.	*Methods*<br>As mentioned in the abstract comments I suggest either adding an "overview" subheading to "data collection" which summarises the timing of baseline assessments, actigraphy, and sleep parameters, or add details to the existing subheadings to ensure readers can easily follow how these all link.	Thank you for the suggestion.<br>We have now added a subheading, **Overview**, under Data Collection to clarify the timeline of study data collection
6.	*Methods*<br>For each data collection item I suggest adding the units which it was coded in, to ensure full statistical transparency and to ensure readers interpret the results accurately. For example, a sentence at the end of each to state that it was recorded in x units, or had a scale of x to y. This was reported for ADL and frailty, but not others.	We have now provided the unit of measure for all the items under **Data Collection,** as recommended.

7.	*Methods*<br>Table 1. These parameters all describe a single day, but the participants were followed for up to 7 days. I imagine the mean value was used, but there are alternative, so this should be directly stated in the methods.	The sleep parameters were averaged across all valid nights within the 7-day study wear-period<br><br>We have now indicated this information within the **Methods** section (under *Sleep Parameters*)

8.	*Methods*<br>Modelling strategy: To align with STROBE I recommend adding more details – (a) that both unadjusted and adjusted regression was performed, (b) listing all 8 sleep outcomes, and (c) listing all exposures. This will help to prepare readers for the results, ensuring they understand accurately, and do not expect anything different. For example, it was stated that sleep efficiency was the primary proxy, wo readers could expect that only this will be modelled.	We have now included all the relevant information, as highlighted by the reviewer, under the **Statistical Analysis** section.<br><br>We have also revised the **Abstract** and the **Methods** to clearly state that the primary outcome measure was sleep efficiency; all other sleep parameters were classified as secondary or exploratory outcomes.
9.	*Methods*<br>Sleep efficiency: This is part analysed as a binary variable (good vs poor sleeper) and part as a continuous (in the main model). I suggest making this consistent, or explaining the rationale for changing it.	Our use of sleep efficiency (SE) in two forms had distinct purposes:<br><br>**Descriptive profile:** For the baseline characteristics tables, participants were grouped into efficient *vs* inefficiency sleep (our primary outcome) using a pre-specified, clinically meaningful SE threshold (detailed in **Methods** section). This binary presentation was used to give readers an immediate, clinically intuitive picture of how characteristics differ between the two groups.<br><br>**Inferential analyses:** For all association models, SE is treated as a continuous variable. Dichotomising SE (a naturally continuous measure) would reduce information and precision and may introduce misclassification; analysing SE continuously provides more accurate and interpretable estimates of association with (mostly) continuous clinical variables.<br><br>We have clarified this rationale in the **Statistical Analysis** section, highlighting that binary grouping of SE is to aid comparison of clinical characteristics; all association analyses are carried out with SE being a continuous variable.

10.	*Methods*<br>Multiple testing: there are over 100 p-values in the results, which greatly increases the likelihood of a significant finding due to chance and undermines the confidence readers can have in the results.<br><br>The authors could account for multiple testing by applying a p-value correction, and/or streamlining the analysis.	We acknowledge the increased risk of Type I error with the multiple testing in the study.<br><br>Incorporating suggestions from Reviewer 2 on a similar concern, we have now stated explicitly (under **Statistical Analysis**) that *p*-values are interpreted descriptively without multiplicity adjustment.<br><br>In addition, we have presented Benjamini- Hochberg adjusted *p*-values controlling the false discovery rate (α = 0.05) in the Supplementary material (**Table S4**).<br>Associations that remained significant after BH correction are indicated with asterisks (**) in (*the new*) **Table 4**[#,†] with accompanying footnote.<br><br>Further, we have removed (previous) Table 4 and the unadjusted model from (previous) Table 5 (and placed in the supplementary material), as recommended by the reviewer.<br><br>We have also advised the readers to exercise caution in interpreting the results, under **Limitation**.<br><br>[#]With the removal of previous Table 4 from the manuscript (in response to Comment No. 10c), previous Table 5 is renamed as **Table 4** in the revised manuscript.<br><br>†We were unable to present the *p*-values in Table 4 (in the main manuscript), due to the tables formatting requirement of the journal (our previous regression table was deemed too large; maximum columns allowed = 9).<br>Instead, we have indicated unstandardised coefficients that were statistically significant with an asterisk (*) in Table 4 and signposted readers to Supplementary **Table S3** for the full regression results.

10a.	<u>For example</u>:<br>Setting a primary outcome. Sleep efficiency was described as a primary proxy but this hierarchy is not applied to the methods where all outcomes are presented equally	Our primary objective was to examine sleep efficiency (SE) in relation to markers of HF severity, functional status and HRQoL, whilst analyses of other sleep parameters (SPT-window, WASO, sleep onset time, wake-up time, SRI, sleep duration and number of sleep interruptions) planned as exploratory.<br><br>We recognise that this hierarchy was not clearly reflected in the original Methods section. We have now revised the text (in **Abstract** and in main **Methods**) to explicitly define SE as our primary sleep parameter of interest and to label analyses involving the other sleep metrics as exploratory.
10b.	Table 3 and the unadjusted results in Table 5 are conceptually making the same comparisons. Unadjusted results also have limited interpretability due to confounding. Could the unadjusted results be kept as an appendix?	We agree that unadjusted models have limited interpretability and therefore have moved the results to the Supplementary material **(Table S2)**, as recommended.<br><br>We present only the adjusted model in the main manuscript (*new* **Table 4**).
10c.	Table 4 reports statistics which are outside of the aims of the study – it compares SE categories against the other sleep outcomes. I am unsure what the reader should take from this – is it that the outcomes tend to align and therefore SE is valid as a primary proxy? I am not sure of the purpose of the p-values and suggest the authors consider if they are needed, either integrating it with the aims more or perhaps moving it to an appendix.	We initially explored the differences in other sleep metrics between participants with efficient *vs* inefficient sleep to identify parameters that might drive the variation in their overall sleep (in)efficiency.<br><br>We recognise, however, that these comparisons lie outside the prespecified primary and secondary objectives of the study.<br><br>We have now moved the table to **Supplementary material** (Table S1) and the corresponding Figure 3 has been removed from the manuscript.
10d.	Streamlining the exposures if there are some with poor variance and therefore weak statistical power.	Following re-examination of the data, all exposures appear to have reasonable variance and therefore have been retained in the regression analysis for completeness.

		We have however, undertaken other measures as recommended by the reviewer to account for multiple testing (as indicated above)
11.	*Results*<br>The results are all very clearly reported, including the tables. All information necessary is reported, and there is a good level of detail in the text. My only minor comments relate to table 5. "model 1 and 2" would be much clearer just stating "unadjusted" and "adjusted". Or even better by splitting them into two tables, for the reasons mentioned above. The N of the models should be reported to align with STROBE.	Thank you for the feedback.<br><br>We have now split (previous) Table 5 into two - 'adjusted' and 'unadjusted' and have moved the 'unadjusted' results to Supplementary material **(Table S2).**<br><br>We have also included the **N** on each model in compliance with STROBE guideline, as recommended.
12.	*Discussion*<br>The interpretation is justified by the findings, and has a good balance between caution and inference. In the limitations I suggest evaluating the risk of confounding bias – the authors adjusted for three confounders, are there any other plausible factors? If not, this is worth also stating, as a strength.	We appreciate the positive feedback.<br><br>Whilst we have accounted for the most common confounders in the study, we acknowledge that the potential effects of other unmeasured factors (such as medication use and sleep hygiene) cannot be excluded.<br><br>We have indicated this as a **Limitation** of the study.

Note: Revisions on the manuscript are indicated in blue font.

References:

1. van Hees, V. T., Sabia, S., Jones, S. E., Wood, A. R., Anderson, K. N., Kivimäki, M., Frayling, T. M., Pack, A. I., Bucan, M., Trenell, M. I., Mazzotti, D. R., Gehrman, P. R., Singh-Manoux, B. A., & Weedon, M. N. (2018). Estimating sleep parameters using an accelerometer without sleep diary. *Scientific reports*, *8*(1), 12975. https://doi.org/10.1038/s41598-018-31266-z

2. American Psychiatric Association. (2020). Diagnostic and statistical manual of mental disorders (5th ed., text rev.). http://doi.org/10.1176/appi.books.9780890425787

3. Andrews, L. K., Coviello, J., Hurley, E., Rose, L., & Redeker, N. S. (2013). "I'd eat a bucket of nails if you told me it would help me sleep:" perceptions of insomnia and its

treatment in patients with stable heart failure. *Heart & lung : the journal of critical care*, *42*(5), 339–345. https://doi.org/10.1016/j.hrtlng.2013.05.003

VERSION 2 - REVIEW

Reviewer **2**

Name **Pacheco, André**

Affiliation **Oslo University Hospital, Department of Research and Innovation, Division of Mental Health and Addiction**

Date **07-Jan-2026**

COI

I thank the authors for their revised manuscript. My previous comments have been satisfactorily addressed. However, one minor point remains in the now revised abstract; please maintain non-causal tone in the concluding parts of the abstract. More specifically, this is regarding ”Sleep irregularity affected HF symptom load, frailty, functional performance and QoL” – to maintain non-causality here, could be rephrased as ”linked to” or ”related to”. Adjusting this to a less causal version would align better with the rest of the otherwise well-written manuscript.

VERSION 2 - AUTHOR RESPONSE

**Reviewer #2**		
1.	I thank the authors for their revised manuscript. My previous comments have been satisfactorily addressed. However, one minor point remains in the now revised abstract; please maintain non-causal tone in the concluding parts of the abstract.<br>More specifically, this is regarding “Sleep irregularity affected HF symptom load, frailty, functional<br>performance and QoL” – to maintain non-causality here, could be rephrased as “linked to” or “related to”.<br>Adjusting this to a less causal version would align better with the rest of the otherwise well-written manuscript.	We thank the reviewer for the feedback.<br><br>We have now rephrased the concluding statement in the **Abstract** to ensure non- causality:<br><br>*“Sleep irregularity was linked to HF symptom load, frailty, functional performance and QoL, while sleep fragmentation was associated with impaired gait speed.”*
